# Microplastic Uptake in Vegetables: Sources, Mechanisms, Transport and Food Safety

**DOI:** 10.3390/toxics13080609

**Published:** 2025-07-22

**Authors:** Zorana Srećkov, Zorica Mrkonjić, Mirjana Bojović, Olivera Nikolić, Danka Radić, Vesna Vasić

**Affiliations:** 1 Faculty of Ecological Agriculture, Educons University, Vojvode Putnika 87, 21208 Sremska Kamenica, Serbia; zorana.sreckov@educons.edu.rs (Z.S.); zorica.mrkonjic@educons.edu.rs (Z.M.); mirjana.bojovic@educons.edu.rs (M.B.); olivera.nikolic@educons.edu.rs (O.N.); 2 Institute of General and Physical Chemistry, University of Belgrade, Studentski trg 12/V, 11000 Belgrade, Serbia; dradic@iofh.bg.ac.rs

**Keywords:** microplastics, nanoplastic, plants, vegetables

## Abstract

Although microplastic pollution has been recognized as one of the major environmental challenges of the 21st century, its toxicological impact on crops, especially vegetables, has attracted limited scientific attention until recently. Vegetables represent a key component of the human diet, making any potential contamination of great importance for food safety. In recent years, an increasing number of studies have been conducted to investigate the interactions between microplastics and vegetable crops. This review aims to synthesize the current knowledge on the sources of microplastics in agroecosystems, the mechanisms of uptake and translocation in plants, and the physiological and biochemical responses induced by micro- and nanoplastics. This work aims to improve the scientific basis for assessing the risk of microplastic contamination by identifying gaps in current understanding and suggesting future research directions.

## 1. Introduction

At the beginning of the 20th century, the first plastic polymer was synthesized. Because of its durability, technical characteristics, low production costs and wide availability, plastic began to be extensively used, eventually becoming the dominant material both in industrial production and in everyday life. However, the excessive use of plastic has led to global plastic pollution, known as synthetic pollution. The accumulation of plastic waste represents one of the greatest challenges facing modern society. Due to various human activities, the amount of plastic waste is constantly increasing, and its pollutants, micro- and nanoplastics, are continuously accumulating in all parts of the biosphere around the world. The steady increase in the concentration of microplastics (MPs) and nanoplastics (NPs) continues despite repeated warnings from the scientific community and ethical advocates about the long-term consequences for ecosystems and human health [1].

Since 1950, the global annual production of plastic has shown a continuous upward trend, reaching 413.8 million tons in 2023 [2]. However, despite efforts to manage plastic waste, projections indicate that by 2050, the amount of plastic pollution released into the environment could range between 66 million tons [3] and 121 million tons [4]. While high- and upper-middle-income countries are expected to reduce their per capita plastic pollution, low-income countries may become increasingly significant contributors [3].

The European Food Safety Agency defines microplastics as particles between 0.1 μm and 5 mm in size, while smaller-diameter particles are classified as nanoplastics [5]. MPs are divided into primary and secondary types depending on how they are formed. Primary MPs include microscopic particles produced in industry, such as microbeads used in personal hygiene preparations, cosmetics and medicines, as well as synthetic fibers released during the production, transport and washing of textiles [6]. In addition, plastic pellets—although not microscopic—are considered primary microplastics because they can directly enter the environment and degrade into smaller plastic particles over time. Secondary MPs are formed by the breakdown of MPs over time [7] through decomposition and fragmentation due to environmental factors, such as UV radiation, exposure to water and mechanical forces [8].

Although plastic is considered a global pollutant, much more research attention was initially paid to the impact of MPs on aquatic ecosystems and aquatic life [9]. More recently, since 2017, researchers have been paying increasing attention to the impact of MPs on terrestrial ecosystems [10], as well as their direct impact on human health due to their entry into the food chain. The current amounts of microplastics in terrestrial ecosystems (in all habitats) are constantly increasing due to various sources, such as the constant use of mulch films in agriculture; irrigation; the use of urban waste as municipal compost for fertilizing agricultural crops; the use of wastewater, both for irrigation and as organic fertilizer; “releases” from wild landfills, mostly located in the immediate vicinity of arable fields or on the arable fields themselves; and natural flows in nature, such as floods, atmospheric intake and so on [10,11,12,13].

Terrestrial plants are affected by a large number of stressors, including micro- and nanoplastics (MNPs). Given the possibility of MNPs entering the food chain through plants, which represents a significant risk to human health, it is very important to study not only the impact of MNPs on living organisms but also their behavior in the environment [1,14]. In addition to the fact that microplastics can enter the food chain through plants, their presence in the soil changes the agroecological conditions in which plants grow. In agroecosystems, where the soil is exposed to intensive agricultural practices, microplastics can affect the physical and chemical properties of the soil, reducing its fertility and nutrient availability. Moreover, the presence of plastic particles can disturb the microbiological balance in the rhizosphere, which can have consequences for plant interactions with beneficial microorganisms. Of particular concern is that vegetable crops, which have short growing cycles and are often grown in greenhouse systems, may be among the first plants through which microplastics enter the food chain [15,16].

The aim of this work is to provide an overview of the latest research on the mechanisms underlying the behavior of microplastics in agroecosystems, with a special emphasis on the impact of microplastics on one of the most important groups of plants used in human nutrition: vegetable crops.

## 2. Materials and Methods

A review of the literature was carried out to collect data on the presence of MNPs in vegetable species. The search involved collecting data on uptake, translocation and potential consequences for plant species, primarily vegetable species.

The search for papers was carried out using the Scopus platform (Elsevier). When selecting the database, the advantages and disadvantages of currently available databases were considered, and the Scopus platform was chosen because it covers a large number of journals [17].

Based on the search terms “microplastics”, “nanoplastics” and “vegetables”, a search query was created for the Scopus database (Table 1). The search was limited to the last 5 years (from 2020 to April 2025), and a total of 260 papers were obtained. After introducing restrictions (Table 1), the number of papers was reduced to 189. However, during the analysis of those papers, another 128 papers were excluded because they did not refer to the presence, uptake or toxic effects of MNPs in vegetables, which was the main goal of this research. In addition, papers that provide a literature review on the presence of MNPs in plant species were excluded. Ultimately, 61 scientific papers were analyzed, and their data are presented to demonstrate the current knowledge on MNPs in vegetable species, including the mechanisms of MNP uptake, the conditions under which this uptake occurs, and the toxic effects of MPs on vegetable species.

In addition to the 61 experimental studies identified through the structured literature search and included in the synthesis, an additional 73 references were selected through targeted searches, citation tracking (snowballing), and expert-based selection. These references were used to provide context, theoretical background, and broader insights into microplastic pollution in agroecosystems, plant–plastic interactions, and related environmental issues.

## 3. Microplastics in Agroecosystems

Microplastics are ubiquitous in the environment. They have been detected in water, soil, air, food and drinking water, indicating their entry into the food chain, which represents a significant threat to human health [1].

### 3.1. Sources of Microplastics in the Soil

Microplastics can enter the soil as a result of various anthropogenic activities and processes in the environment. They are transmitted horizontally and vertically through the soil: horizontally by wind and water and vertically by water or soil organisms [18]. Degradation processes occur through microbiological or physico-chemical means. The primary sources of microplastics are agriculture [10,11,12,13], waste management [11,12] and atmospheric deposition [10,12], which significantly contribute to the accumulation of these pollutants in terrestrial ecosystems. Understanding the original sources of MPs is of great importance for establishing their impact on the environment and human health.

#### 3.1.1. Agriculture as a Source of Microplastics

One of the most significant sources of MPs in the soil is agriculture. It is well known that years of unsustainable land use in agriculture have led to the degradation of a large percentage of the world’s land to varying degrees [19]. In order to overcome the challenges facing today’s society due to diminishing soil fertility, various sustainable techniques for increasing soil fertility have been proposed. One such technique is the application of organic fertilizers, which, in addition to providing nutrition for plants, also increase the content of organic matter in the soil and thus improve its physical, chemical and biological properties, i.e., soil fertility. On the other hand, with the continual increase in the population and urbanization, the amount of wastewater and municipal waste is also increasing. In order to develop sustainable cities, it is necessary to find a sustainable solution for the ever-increasing wastewater, and one of the solutions is to use the sewage sludge resulting from wastewater treatment as an organic fertilizer to increase soil fertility.

It has been established that sewage sludge can significantly increase the content of organic matter in the soil, as well as nitrogen and phosphorus, the most important macroelements [19,20,21,22], and also has a positive effect on the water regime of the soil [23]. However, in addition to all the benefits of sewage sludge, negative aspects of its use have also been identified. Due to the long-term use of sewage sludge, heavy metals can accumulate in the soil [24,25], which increases the possibility of their uptake by plants and entry into the food chain, posing a risk to human health. Heavy metals negatively impact soil microbiological activity, reducing the number and diversity of microorganisms, which in turn diminishes soil fertility [25]. Beyond heavy metal accumulation, recent research indicates that sewage sludge used as fertilizer introduces microplastics into the soil, which become plant-available and enhance heavy metal uptake [11,12,13].

In addition to sewage sludge, compost is a significant source of plastic in agricultural soils [13]. In particular, it was confirmed that compost can contain microplastics that originate from composted plant material that is not destroyed in the composting process, and the use of such compost can lead to the accumulation of microplastics in the soil [26].

Plastic mulch films are also a significant source of microplastics and are used intensively, especially in vegetable production. Mulching is an agricultural technique with great benefits, not only for the final yield of agricultural crops but also for the quality of the soil and the fruits themselves. Mulching involves covering the soil with different materials, which reduces water evaporation, thus positively affecting the water regime of the soil and plants, and reduces the need for irrigation, helps control weeds and regulates soil temperature conditions [27], while also decreasing the possibility of aeolian erosion. All these features enable more sustainable agricultural production with satisfactory yields, which makes the mulching process popular with farmers. However, during vegetation, atmospheric and human influences cause damage to the plastic mulch film—one of the most commonly used mulch materials—and smaller fractions are released into the soil [11]. Moreover, removing plastic films, especially smaller fragments, at the end of the growing season is an extremely expensive and laborious job [28] and often not carried out, which leads to the accumulation of plastic fragments (micro and nano) in the soil [27,29], the pollution of the agroecosystem with microplastics and the entry of micro- and nanoplastics into the food chain [30], which can directly affect people’s health.

In order to reduce the negative impact of plastic mulch film on the agroecosystem, biodegradable mulch films have been increasingly used in recent years, which significantly reduce plastic pollution in soil because soil microorganisms facilitate their decomposition [31]. However, this is not a permanent solution because even biodegradable foils are damaged and release plastic particles into the soil under the influence of atmospheric precipitation, UV radiation and human activity during the growing season [32].

Plastic mulch films are not the only agricultural products created from plastic. Plastic is also used to produce greenhouses and low and high tunnels [33], anti-hail nets, packaging for pesticides, ropes for tying plants, nets for supporting plants, irrigation systems [29] and other auxiliary products that are commonly used in vegetable production. In addition, some pesticides and fertilizers may contain microplastic particles that are added as carriers of active substances or as part of the formulation, so their application introduces microplastics directly into the soil.

#### 3.1.2. Waste Management and Atmospheric Deposits as a Source of Microplastics

In addition to agricultural production, improper and illegal waste disposal [34] and “leaks” that occur in wild landfills and poorly controlled legal landfills, as well as atmospheric deposits, are considered large sources of MNPs in agroecosystems. Once MNP particles reach the soil surface, because of their size and mobility, they accumulate in the surface layers of the soil.

### 3.2. Influence of Microplastics on Soil Characteristics

MPs affect the physical, chemical and biological properties of the soil and thus indirectly affect the growth and development of plants [35]. The presence of MPs can disrupt soil structure, affecting water retention and nutrient availability [36], which can have negative consequences for soil microorganisms, plant nutrition and overall ecosystem health [12]. In addition, the presence of MPs (0.4% (*w*/*w*)) reduces the volumetric density and soil pH, causes changes in aggregate size in the soil and masks the real carbon content in the soil, all of which leads to soil degradation over time, reduces soil fertility and negatively impacts plant growth and development [37]. Although the added plastic accounted for only 0.4% *w*/*w*, the study demonstrated that even such low concentrations can significantly alter soil physical structure, chemical characteristics, and microbial activity, likely due to the high surface area, chemical reactivity, and persistence of microplastics in the soil environment.

Microplastics change the soil pH value. The change can occur in either direction, but most studies that dealt with the impact of microplastics on soil properties concluded that they lower the pH value [38,39,40], thereby increasing soil acidity. The addition of microplastics significantly reduced soil pH, particularly at higher concentrations of 130 μm MPs, and this change in pH directly affects the availability of certain nutrients to plants [39], directly affecting their growth and development. Also, the addition of polyethylene microplastics (PE-MPs) led to a measurable decrease in soil pH values in cadmium-contaminated soils. Although the original study reported percentage reductions (0.66% to 2.10%) with increasing PE levels, no absolute pH values were provided [40]. A change in pH also affects the mobility of various pollutants, especially heavy metals [41]. MPs are small but have a large surface area, which allows them to absorb various organic and inorganic pollutants in the soil [11] and to serve as media that enhance the uptake of pollutants by plants [42].

## 4. Soil–Plant System

### 4.1. Mechanisms of Microplastic Uptake

As previously mentioned, land plants are directly influenced by water, soil and air. Thus, MNP uptake does not occur through only one mechanism, and several mechanisms of absorption via the roots and leaf surface have been identified [9].

Plants can take up microplastics through their roots through several mechanisms. One mechanism of MP entry is penetration through cracks. Specifically, when the root grows, small cracks can form on its surface, especially in the zone of elongation and cell differentiation. These cracks are caused by mechanical stress, growth in solid soil, the activity of microorganisms or even interactions with other particles in the soil.

Microplastic particles present in the soil can accumulate in these cracks and passively penetrate the interior of the roots together with water and dissolved substances. After entering through cracks, the mentioned particles can move through the apoplast (intercellular spaces) or endodermis, potentially reaching the central cylinder (where the conducting bundles are located), from where they can be transported to the aerial parts of the plant. This mechanism is particularly significant because it allows MPs to enter plants that do not have specialized structures for the intake of larger particles, thereby increasing the risk of their entry into the food chain [43].

The second mechanism occurs upon the initial contact of MPs in the soil with the plant cell membrane. This contact can be passive (if particles are present in solutions) or active, when the plant reacts to foreign particles through recognition mechanisms. Yue et al. [41] explained how endocytosis works in the context of microplastics. Specifically, when a microplastic particle (100 nm to 1000 nm) approaches the cell membrane, the cell can “recognize” this particle as a substance that needs to be taken in. The cell membrane retracts (invaginates) around the particle and forms a vesicle that then “encloses” the particle within it. After the vesicle is formed, it separates from the outer part of the cell and enters the cytoplasm. Microplastic particles that enter the cell can be transported to different parts of the cytoplasm, where they can accumulate or even lead to mechanical damage.

In addition to the uptake of micro- and nanoplastics through the roots, plants can also take them up through the leaves. There are two assumed routes of MNP entry through the leaf: through the cuticle and through the stomata. According to research by Lian et al. [44], Luo et al. [45] and Sun et al. [9], absorption of MNPs by leaves mainly occurs through stomata from the air, reaching the apoplastic pathway before being transported throughout the plant.

To estimate the proportion of MPs absorbed by plants through the leaves and determine how they are transported from the leaves to the roots, Sun et al. [9] foliarly treated corn (*Zea mays* L.) seedlings with two types of plastic (carboxyl-modified polystyrene nanoplastic (PS-COOH, 24.0 ± 2.2 nm) and amino-modified polystyrene nanoplastic (PS-NH_2_, 22.0 ± 1.5 nm)) and found that both types successfully accumulated on the leaf surface. Positively charged PS-NH_2_ showed greater association with the leaf surface (since the cell walls are negatively charged) compared to negatively charged PS-COOH. Nanoplastics are absorbed mainly through stomata into the vascular system of corn and are further transported to the stem through the xylem and phloem. However, no significant presence of NPs in the roots was found. They assume that small NP particles can easily pass through the vascular bundle to the roots, but larger NP aggregates move with difficulty and are therefore absent in the roots. This suggests that the translocation of nanoplastics within the plant is size-dependent, with smaller particles more likely to reach the roots than larger NP aggregates, due to size-limited movement through vascular tissues.

### 4.2. Reaction of Plants to Microplastics

The impact of microplastics on plants has been proven in several works [9,33,44,46,47]. Plants vary in their reactions to the toxic effects of MPs, from changes in growth and development phases to changes in morphology, all of which are conditioned by changes at the level of physiological and biochemical processes. All these changes can affect the yield of agricultural crops, emphasizing the need to study this problem.

#### 4.2.1. The Influence of Microplastics on Seed Germination

When scientists began to focus on the impact of MPs on terrestrial ecosystems and terrestrial plants, one of the stages of development that they paid particular attention to was seed germination. This is logical because germination is the first stage of development that affects the other stages, and any stress during germination can negatively affect the growth and development of plants, and thus the crop yield.

Plastic particles can affect seed germination in different ways. Because of their high adsorption capacity, microscopic particles can accumulate on the surface of seeds, physically blocking the pores and thus preventing the uptake of water, which is necessary for seed swelling and germination [48]. MPs do affect the physical absorption of water by seeds, which slows down the swelling and germination process itself, but also negatively affect the biological processes in the seeds [49,50,51,52].

The influence of MPs on seed germination has been confirmed in a large number of studies [50,53,54,55,56,57,58,59,60,61]. However, in several other studies, no significant impact of MPs on germination was found [62,63,64,65]. Huang et al. [47] explain this by suggesting that the influence of MPs on seed germination depends on the type and concentration of plastic and the plant species.

#### 4.2.2. Changes in Morphological Features

Under the influence of microplastics, whether in the soil, water or air, various changes occur in the roots and aboveground mass of the plant [46,53,66,67,68].

Lozano et al. [46] found that the impact of MPs on plant properties and the physical and biological properties of soil depends more on the shape and type of MPs than on their concentration (0.1, 0.2, 0.3, and 0.4%). Studying the influence of different forms of MPs with different chemical compositions on the properties of wild carrots (*Daucus carota*) and on the properties of the soil, they found that microplastics in the soil led to a 46% increase in root mass and as much as a 48% increase in shoot mass on average. The greatest increase in shoot and root mass was recorded with an increased concentration of MPs in the film type (60%), while MPs in the fiber type resulted in the smallest increase in shoot (27%) and root (6%) mass. The authors explain this as an indirect effect of MPs through changes in soil properties. Fibers decreased soil aggregation more than films (29% vs. 25%) and reduced microbial activity at higher concentrations, while low concentrations of PA fibers increased microbial activity. Soil aggregation was quantified as the proportion of water-stable aggregates (WSA), measured using the method described by Kemper and Rosenau (1986), which expresses the structural stability of soil against slaking and mechanical disturbance in water [46]. Specifically, microfibers reduce soil bulk density, which causes an increase in soil porosity and a change in the air regime of the soil (the soil becomes more transparent, or “lighter” [69]). It is known that in “lighter” soils, it is easier to penetrate and accumulate in the root system, which directly improves the utilization of nutrients and water, especially those in deeper layers, all of which leads to greater biomass and an increase in the mass of both roots and shoots. In addition to this, the possibility of creating a mycorrhizal bond, which is known to have a positive effect on biomass, also increases.

#### 4.2.3. Changes in Physiological and Biochemical Processes

Plants react to MPs in the soil through various physiological mechanisms that allow them to adapt to stressful conditions. These responses include changes in water and nutrient absorption, the activation of antioxidant systems and growth regulation [46,54,70,71].

From a biochemical point of view, the response of plants to MPs in the soil can be observed through the activation of antioxidant enzymes, such as superoxide dismutase (SOD), catalase (CAT) and peroxidase (POD), which neutralize reactive oxygen species (ROS) caused by toxic substances. These enzymes help reduce oxidative stress that can damage cellular structures [70]. Similarly, the presence of MPs can cause changes in the primary and secondary metabolism of plants. Plants under stress often produce more secondary metabolites, such as phenolics and flavonoids, which play a role in stress protection. This can increase their resistance to the harmful effects of MPs and the pollutants they carry [72].

Ren et al. [73] summarized research on the impact of micro- and nanoplastics on vascular plants at the metabolic and molecular levels. Having reviewed and summarized a large number of papers, they concluded that the influence of MNPs on vegetative growth and plant reproduction processes can be direct and indirect, but that the mechanisms of influence themselves have not yet been clearly defined and clarified. Plants can acquire MNPs through the roots or through the leaves, causing various physiological and biochemical changes in the plants themselves. The authors especially emphasized changes in carbon and nitrogen metabolism, which are necessary for the growth and development of plants, as well as changes in the synthesis of amino acids. The presence of plastic particles also caused changes in gene expression, primarily activating genes that help protect plants from stress and pathogen attacks. In terms of physiological changes, they focused on summarizing results on the impact of MNPs on seed or spore germination, photosynthesis and oxidative stress, as well as on the toxic effects of microplastics at the cellular level, all of which affect the health of plants in the long term.

### 4.3. Microplastics in Vegetables

Vegetable species are among the most important foods in the human diet, so it is not surprising that researchers studying the impact and accumulation of MPs in plants began to focus on this group. In recent years, several studies have been devoted to this group of plants (Table 2). However, in most of these studies, vegetable species are used as model plants for research, and very little effort is devoted to examining the levels of microplastics in vegetables already on the market [5].

**Table 2:** Effects of MNPs on vegetables.

Vegetables	Effects	Reference
Broccoli (*Brassica oleracea var. italica* Plenck.)	Decrease in growth, chlorophyll content, photosynthesis, and nutrient uptake	[74]
	Affected growth parameters, lipid peroxidation rate, and phytochemicals.	[75]
Cauliflower (*Brassica oleracea var. botrytis* L.)	Alteration in photosynthetic pigments and antioxidant enzyme activity	[76]
Carrot (*Daucus carota* L.)	Deformation of cell walls	[77]
Chinese flowering cabbage (*Brassica rapa var. parachinensis* L.)	Reduction in chlorophyll content, photosynthetic rates, plant growth and nutritional quality	[78]
	Delayed the seed germination process, disruptions in oxidative stress, osmoregulation, photosynthetic function, and elemental reservoirs	[79]
	Decrease in plant growth	[80]
Cucumber (*Cucumis sativus* L.)	Alteration in nitrogen and carbohydrate metabolism; decrease in photosynthetic capacity.	[47]
	Increase in root activity, MDA, proline and soluble protein content; decreased Mg, Ca and Fe levels in fruits	[81]
Field mustard spinach (*Brassica rapa var. perviridis* L.)	Reduction in fresh and dry yield	[82]
Garlic Chives (*Allium tuberosum* Rottler ex. Spring)	Reduction in chlorophyll content and photosynthetic rates	[78]
Leaf mustard (*Brassica juncea* L.)	Inhibited nutrient uptake and promoted early flowering in plants, increased ROS in leaves. Reduced photosynthetic parameters.	[83]
Lettuce (*Lactuca sativa* L.)	Alteration of plant growth and root activity, increase in cadmium concentration	[84]
	Decrease in shoot fresh and dry weight, photosynthetic activity and pigment content, increase in leaf water content	[85]
	Decrease in plant growth	[86]
	Increase in expression of flavonoid biosynthesis genes and flavonoid content	[68]
	Alteration in plant growth and photosynthetic pigments; increase in antioxidant enzyme activity	[87]
	Significant impact on metabolic pathways; increase in plant fresh and dry weight	[88]
	Alteration in plant growth and antioxidant enzyme activity	[89]
	Alteration in root morphological parameters and nutritional quality	[90]
	Reduction in plant growth; increase in photosynthetic pigments, and enhanced cadmium toxicity	[91]
	Increase in plant aboveground biomass, nitrogen use efficiency and chlorophyll content	[92]
	Decrease in plant biomass and chlorophyll content, alteration in antioxidant enzyme activity	[93]
	Significant impact on plant biomass and oxidative stress indicators	[94]
	Reduction in plant growth, and chlorophyll content, increase in nitrate content and Cd accumulation, alteration in cell membrane integrity and metabolite levels, impaired antioxidant defense capacity,	[95]
	Inhibition of seed germination, reduction in early plant height	[96]
	Accumulation of heavy metals in roots Increase in heavy metal concentration	[97]
	Reduction in plant growth and total phlorophyll content; increase in antioxidant enzyme activity	[98]
	Reduction in plant growth total antioxidant capacity and essential amino acid biosynthesis, alteration in micronutrients and chlorophyll content, decrease in	[44]
	Reduction in fresh weight, reduction in root and shoot length, induction of oxidative stress, impaired water and nutrient transport	[99]
	Reduction in growth and chlorophyll content	[100]
	Inhibition of root growth and seed germination	[60]
	Decreased photosynthetic parameters and total chlorophyll content, increased hydrogen peroxide, malondialdehyde levels and nitrite content	[101]
	Reduction in root and shoot biomass, induction of oxidative stress	[102]
	Alteration in Cd accumulation	[103]
	Reduction in plant growth, induction of oxidative stress	[104]
Melon (*Cucumis melo* L.)	Decrease in germination potential and plant growth	[53]
Pak choi (*Brassica rapa*. ssp. *Chinensis* L.)	Decrease in plant growth; alteration in antioxidant enzyme activity and cell morphology	[105]
	Alteration in phenolic compounds; increase in phytohormone derivate content	[33]
	Alteration in chlorophyll content, nitrogen use efficiency and overground and belowground mass	[92]
	Increase in total leaf carbon and nitrogen content, relative water content and plant growth; decrease in chlorophyll content, transpiration rate and stomal conductance	[78]
	Reduction in plant growth and chlorophyll content, alteration of soluble sugar content	[106]
	Alteration in metabolic activities	[107]
	Reduction in shoot and root length, plant biomass and chlorophyll content	[108]
	Inhibition of plant growth, reduction in fresh and dry shoot weight, alteration of chlorophyll, soluble protein and vitamin C content	[109]
	Reduction in shoot length and fresh weight, SPAD values, increase in SOD, POD and CAT activities	[110]
	Reduction in leaf number, fresh weight, total protein content, photosynthetic pigment content, alteration in root activity and antioxidant enzyme activity	[111]
Pepper (*Capsicum annuum* L.)	Disruption in amino acid metabolism	[112]
	Decrease in plant growth, fruit yield and antioxidant enzyme activity, increase in MDA content	[113]
Potato (*Solanum tuberosum* L.)	Alteration in photosynthetic content and antioxidant enzyme activity	[76]
Potherb mustrad (*Brassica juncea var. multiceps* L.)	Disruption in carbohydrate, amino acid and lipid metabolism; yield reduction; increase in Fe/Mn content and antioxidant enzyme activity	[114]
Radish (*Raphanus sativus* L.)	Alteration in antioxidant enzyme activity, germination rates and relative root lengths	[76]
	Alteration in plant growth	[115]
	Decrease in plant growth; alteration in antioxidant enzyme activity; increase in chlorophyll a content;	[116]
	Decrease in plant growth and nutritional values, increase in MDA and anthocyanin content	[75]
	Decrease in germination percentage, alteration in plant growth and antioxidant enzyme activity	[60]
	Reduction in plant growth, increased variability in plant growth	[117]
Red amaranth (*Amaranthus tricolor* L.)	Alternation in plant growth; reduction in nitrogen, phosphorus and potassium content; increase in germination parameters and cadmium phytotoxicity	[118]
Spinach (*Spinacia oleracea* L.)	Alteration in plant growth; reduction in membrane stability index and chlorophyll content; increase in proline, protein and soluble sugar content and antioxidant enzyme activity	[109]
	Reduction in chlorophyll a and b and ascorbic acid content and antioxidant enzyme activity; increase in carotenoid; alteration in antioxidant enzyme activity	[76]
Water spinach (*Ipomea aquatica* F.)	Oxidative stress induced	[119]
	Reduction in plant growth	[120]
	Reduction in plant growth and chlorophyll a content	[121]

#### 4.3.1. Type and Form of Microplastics in Vegetables

The phytotoxic effects of MPs on plants vary significantly depending on their size, shape and polymer composition, as these properties influence the particles’ mobility, uptake pathways and interaction with plant tissues [48].

The most common type of MNPs found in vegetable species is polystyrene (PS) [5]. PS-type polymers have been found in the roots of carrot (*Daucus carota* L.) [77], radish (*Raphanus sativus* L.) [60], onion (*Allium cepa* L.) [122], lettuce (*Lactuca sativa* L.) [60], pea (*Pisum sativum* L.) [123] and tomato (*Solanum lycopersicum* L.) [124], as well as in the stem of pea [123].

Oliveri Conti et al. [125] found microplastics in peeled carrots, whole lettuce and broccoli, but the type of polymer was not identified. Rajendran et al. [126] examined fruits and vegetables purchased at the market and found plastics in eggplants and potatoes. They found polyethylene (PE) and high-density PE plastics in the eggplants. In potatoes, they found plastic particles of nylon. Although vegetable species have been included in research testing the toxic effects of MNPs, very few studies have actually examined the presence of MNPs in vegetables that are on the market. Most of the research has focused on the mechanisms of MNP uptake by vegetable species, as well as on the types of reactions of vegetable species to the phytotoxic effects of plastic particles.

Canha et al. [127] included lettuce bought in micromarkets in their research and found MPs in lettuce leaves, and the MP concentration was at the same level as in lettuce grown in rural areas. Aydın et al. [128] examined the content of MPs in fruits and vegetables found in markets, and MPs were detected in all the examined samples. The highest number of particles was found in tomato samples (3.63 particles g^−1^), followed by cucumber (3.60 particles g^−1^) and onion (2.60 particles g^−1^), and the lowest number was found in potatoes (17 particles g^−1^). In addition to the number of MP particles, they also studied the shape, color and type of MPs found in fruits and vegetables. The largest number of samples contained MP particles that were black (45.5% of samples), followed by gray (17.9% of samples), white (16.5% of samples), blue (7.8% of samples) and red (6.1% of samples), with green MPs found in the fewest samples (4.5% of samples). The most common form was a fragment, followed by a fibril, and the least common form was a film. In terms of particle size, 86.1% of the samples ranged from 0.1 µm to 1 mm, and 13.9% from 1 to 5 mm. Low-density polyethylene (PE) was found in 60% of the samples, and polypropylene (PP) and polyethylene terephthalate in 20% of the samples.

#### 4.3.2. Mechanisms of Uptake of Micro- and Nanoplastics by Vegetables

Vegetables can absorb MNPs from soil and irrigation water [33,60,77,117,125,126].

Plastic particles primarily enter through the roots in the rhizoderm zone but are then transported through the xylem [129]. Zytowski et al. [33] studied the uptake, translocation and accumulation of different types of NPs (polymethyl methacrylate; PS—polystyrene) in four vegetable species: three representatives of dicotyledonous plants (pak choi, radish and tomato) and one representative of monocotyledonous plants (asparagus). They found that pak choi took up fluorescent NP particles through the roots, after which they were transported, primarily through the xylem, from the roots to the stem and leaves, and accumulated in the intercellular space.

In their research, Wang et al. [86] also confirmed the uptake of MPs by roots in lettuce (*Lactuca sativa* L.), as well as the translocation of particles within the intercellular space and conducting tissue. Absorption was higher under conditions of higher soil moisture, especially in soil with a higher sand content and a lower clay content. Studying the influence of differently charged polystyrene particles of size 80 nm and 1 μm on the growth and development of water spinach (*Ipomea aquatica* F.) (by adding the particles to the water solution in which the plants were grown), Zhao et al. [119] found micro- and nanoparticles in the roots, leaves and stem. The highest concentration was in the roots, rhizodermis and central (conductive) cylinder, while in the stem and leaves, the particles were concentrated in conductive bundles. Zhao et al. [120] investigated the uptake of polystyrene (PS) microplastic particles by water spinach using a mixed suspension containing both 200 nm and 1 μm spherical PS beads. For the first time, they simultaneously observed the presence of both particle sizes attached to the cell walls of vascular tissue in the roots. The authors hypothesized that the smaller 200 nm particles caused deformation of the root epidermal cells, thereby facilitating the entry of larger, 1 μm particles, which were also detected in the intercellular spaces. The uptake of polystyrene-type plastic particles of different sizes and their translocation into the stem and leaves were confirmed in research by Hua et al. [93]. They studied the influence of MPs of two sizes (100 nm and 500 nm) on morphological, physiological and biochemical processes in lettuce and found that lettuce took up particles of both sizes via the roots and translocated them to the stem and leaf via the conduction system, thus proving that this is one of the ways that MPs are translocated from the roots to leaves. They also found a large number of MPs on the surface of the roots, even after ultrasonic cleaning, as well as microplastic particles in the gaps of the lateral roots, which confirms the theory that the “growing area” of the lateral roots is one route of microplastic uptake.

After being absorbed through the roots, nanoparticles (NPs) can be transported to the leaves. This has been confirmed by research conducted by Xu et al. [68], Castan et al. [130] and Li et al. [81], who found that microplastics are translocated not only to the leaves but also to the flowers and fruits of cucumber plants (*Cucumis sativus* L.).

In addition to uptake through the roots, plastic particles can also be absorbed through the leaves, from where they also, like those absorbed by the roots, move to other organs [9,33]. Lian et al. [44] investigated the effect of the foliar application of nanoplastics on plants. Young lettuce plants were foliarly treated with two solutions of polystyrene NPs (PSNPs) three times over 21 days. After 21 days, measurements were made and analyses were performed to determine whether there were significant changes in the growth and development of plants due to the effect of NPs. Properties that were used as an indicator of changes in plant growth showed significant changes due to exposure to NPs. Plants exposed to NPs were less developed compared to control plants, and dry mass and plant height were significantly reduced at concentrations of 0.1 mg/L (by 14.3%, and. 27.3%, respectively) and 1 mg/L (23.2%, and 24.4%, respectively). In addition, the higher concentration of NPs led to a significant reduction in the leaf surface and the content of pigments (chlorophyll and carotenoids). The dry mass ratio of shoots and roots, the net rate of photosynthesis, water use efficiency and stomatal limitation were also significantly reduced. Contrary to all these reductions, exposure to NPs significantly improved stomatal conductance and transpiration rate. Overall, the exposure of young lettuce seedlings to NPs significantly inhibited plant growth. Wang et al. [98] also studied the possibility of the uptake of microplastics through the leaves and found that after foliar application of a microplastic solution, there was an accumulation of microplastics in lettuce leaves, indicating the possibility of microplastic uptake through the stomata and cuticle.

Huang et al. [47] found that with the foliar application of polystyrene NPs on cucumber (*Cucumber sativus* L.) seedlings, the NPs accumulated in the plant, which resulted in changes in gene expression and metabolic pathways, both of which affected the growth and development of cucumber plants. The effect varied significantly depending on the concentration of NPs, with higher concentrations leading to more pronounced impacts. In addition to studying the uptake of NPs via roots, Zytowski et al. [33] also studied the uptake and translocation of NPs when applied foliarly. They found that in addition to pak choi (*Brassica rapa* L. ssp. *chinensis*) absorbing NPs through the roots, it also absorbed them through the leaves, after which they were translocated through the vascular bundles into leaves, mainly via transpiration.

Ilyas et al. [78] found NP uptake through and accumulation in the leaves of four types of leafy vegetables (pak choi, Chinese flowering cabbage, garlic chives and amaranth). They found that NP particles are mainly located in the epidermal tissue around the stoma and are not in the cuticle (epicuticular wax), which suggests the uptake of nanoplastics through the stoma. After the foliar application of MNP particles in different concentrations, Li et al. [131] did not find a significant change in the mass of fresh aboveground mass in lettuce, but they found changes in the content of chlorophyll, as well as an increase in soluble proteins and sugars and a reduced concentration of nitrates in lettuce leaves. Jiang et al. [132] also found that lettuce took up foliarly applied NPs via stomata, which translocated to the roots over time.

Zytowski et al. [33] found that after MNPs enter the plant, they accumulate in the intercellular space of the tissue, which indicates that they accumulate in the edible parts of the plant and thus enter the food chain. In addition, Wang et al. [86] found microplastics in almost the entire leaf, which represents a greater potential danger to human health.

#### 4.3.3. Conditions Affecting the Adoption of Microplastics

The phytotoxicity of MNPs depends on a large number of factors, such as the size and shape of particles, the type of microplastic [111] and the growing conditions [76,96], as well as the plant species and length of exposure to microplastics [48].

Gong et al. [60] found that the manifestation of the toxic effects of MPs depends on the size and dose of the particles, as well as the plant species exposed to the effects of plastic particles. They studied the impact of polystyrene-type NPs and spherical MPs on four plant species (lettuce, radishes, wheat and corn) and found that lettuce is the most sensitive to the toxic effects of MNPs. Of the four tested species, only lettuce showed a statistically significant decrease in the germination index. Under the influence of NPs, the germination index decreased by 23.8% at the low dose and 36% at the high dose. In the case of MPs, the reduction was 18.2% at the low dose and 29.3% at the high dose. Moreover, in lettuce, significant changes were observed in the dry mass of shoots and roots, the length of roots and the ratio of shoots and roots. Significant changes in the shoot-to-root ratio, root dry mass and root length were also recorded in corn, as well as in the shoot-to-root ratio in radish.

Zhang et al. [96] examined the influence of six concentrations of MPs (0.0%, 0.1%, 0.5%, 1.0%, 2.0% and 5.0%), specifically low-density polyethylene MPs (LDPE-MPs), of different particle sizes (75–2000 μm) on the germination and morphological characteristics of lettuce. They found that significant changes in the morphology of lettuce occurred at medium (0.5% and 1%) concentrations of MPs, and that there was a statistically significant reduction in plant height. They also monitored changes in relation to the length of exposure to the toxic effects of MPs and found that a statistically significant change in the height of the plant occurred after 20 days of exposure, while after a longer period of exposure (28 days), there was a change in the height of the plant, but this change was not statistically significant. The percentage of germination decreased. This reduction was statistically significant compared to the control variant for all concentrations of MPs, except for the highest concentration (5%). MP may accumulate in large amounts between the pores of the seed epidermis, blocking water absorption and delaying germination. This effect might be concentration-dependent, with moderate concentrations causing significant disruption, while higher concentrations may not exacerbate the blockage further or may trigger compensatory mechanisms in the seeds. This research indicates that the medium concentration of MP (0.5% and 1.0%) decreased soil moisture and temperature, and increased nitrogen loss, which negatively impacts germination and early growth of lettuce.

A change in the degree of germination in the presence of different concentrations (0 g/kg, 1 g/kg, 2 g/kg, 4 g/kg, 8 g/kg and 16 g/kg) of MPs was observed by Li et al. [53]. They observed a significant decrease in the germination rate at medium concentrations (2 g/kg and 4 g/kg) and in the germination index at all applied concentrations of MPs compared to the control. No statistically significant differences were found in the seedling mass and radicle length compared to the control, while there was a statistically significant increase in root fork mass at concentrations of 1 to 4 g/kg and root tip mass at concentrations 1 g/kg, 2 g/kg, 4 g/kg and 16 g/kg.

Examining the uptake and translocation of polystyrene-type NPs (PS-NPs) by water spinach at different concentrations (0.5 mg/L, 5 mg/L and 10 mg/L) of NPs, Gao et al. [121] found that there was no uptake of NPs by the plant after a short-term exposure to NPs (10 days), which were only “hooked” to the rhizodermis of the roots. However, although the NPs were not taken up and translocated in the plant, the high concentration of NPs caused changes in the morphological properties of water spinach. There was a highly significant decrease in the fresh mass of the plant and a significant decrease in the length of the shoot and roots. The content of chlorophyll a gradually decreased as the concentration of NPs increased, but the difference was not significant, nor was the change in the content of chlorophyll b. The activity of SOD (superoxide dismutase) and CAT (catalase) decreased as the concentration of NPs increased, and at the highest NP concentration, the difference was statistically significant, while the activity of POD (peroxidase) showed no statistically significant response to NP exposure, even at the highest concentration tested.

Wang et al. [104] examined the influence of six different concentrations of microplastics on the growth and development of lettuce and found that they significantly reduced plant growth. Measurements were made after 6, 12 and 18 days, and all measurements showed a decrease in plant height, root length, number of leaves and fresh weight of the plant and roots relative to the control. After 6 days of measuring enzyme activity, SOD (superoxide dismutase), GR (glutathione reductase), MDA (malondialdehyde) and CAT (catalase) activity in the roots continuously increased with increasing MP concentration, while after 18 days, the activity of the investigated parameters (SOD, CAT, APX, GR, MDA) increased to a concentration of 30 mg/L and then started to decrease. In the leaves, after 12 and 18 days of measurement, the activity of SOD, APX (ascorbate oxidase) and GR continuously increased, while the activity of MDA increased continuously after 12 days; after 18 days, it increased to a concentration of 40 mg/L, after which it decreased.

In addition to the type, shape and size of microplastic particles, soil properties can influence the uptake of microplastics. Meng et al. [133] studied the influence of three types of microplastics (PS—polystyrene; ABS—acrylonitrile butadiene styrene; PVC—polyvinylchloride) without additives (MP) and with additives (MPA—BA, PA, VA) in two types of soil with different chemical properties on the mobility and uptake of metals and their individual and joint effects on the microbiological community and the growth and development of radish (*Raphanus sativus* L.). They found that the presence of MPs, both without additives and with additives, had a significant effect on the growth of radishes. In soil with a lower pH value, there was a decrease in the germination rate, root length and seed germination index, as well as a 14% and 17% decrease in the activity of SOD and the content of MDA, respectively, while the activity of POD increased by 14%. In soils with higher pH values, SOD and POD activity increased by 4–9%, and root length increased by an average of 10%.

Zhang et al. [88] studied the effects of four different sizes and three different concentrations of MNPs on changes in the morphological properties of lettuce. They found that, individually, different particle sizes and different concentrations of MNPs significantly influenced the increase in fresh and dry aboveground mass of lettuce, while fresh root mass was most influenced by the joint action of different concentrations and different particle sizes. Li et al. [108] investigated the effect of two types of microplastics in two different types of soil on the growth and development of pak choi, as well as the behavior of Cd in the soil and its accumulation by the plant in the presence of different types of MPs. They found that polylactic acid (PLA) MPs significantly reduced the fresh weight of the plant (1.77% and 23.26%), shoot length (3.54% and 5.64%), root length (5.65% and 7.75%) and chlorophyll content (2.08% and 2.34%), while polyethylene (PE) MPs did not significantly affect the morphological characteristics of the plant.

Wang et al. [86] examined how, in addition to the size of the microplastic particles, soil moisture and texture influence the intensity of uptake and translocation. They used lettuce as a model plant and studied plant height, leaf length, root length and diameter, chlorophyll index, photosynthetic rate, intercellular carbon dioxide concentration, stomatal conductance and transpiration rate. Three different irrigation regimes (5, 10, and 15 mL of water applied every two days) and two different soil structures (ST1 with 23.67% sand, 24.41% silt, and 51.92% clay; ST2 with 44.09% sand, 30.41% silt, and 25.50% clay), as well as their combinations, were tested. In order to determine the absorption and translocation of plastic particles, fluorescent microparticles of size 100 and 200 nm were used, and in order to confirm absorption and determine the potential negative impact of MPs on plants, they used large, unmarked polystyrene plastic particles (<149 µm). They found that the presence of MPs had a negative effect on the growth of both aboveground and underground parts of the plant, and this effect increased as the size of the plastic particles decreased. They also found that the absorption of microplastics was significantly higher compared to earlier research that examined this issue, and they attributed this difference to the length of time the plants were exposed to the toxic effects of microplastics. Specifically, in contrast to the research of other authors, Wang et al. [86] measured the content of MPs in lettuce plants after 60 days of vegetation, which approximates the conditions of production.

Maryam et al. [82] also concluded that the length of exposure to microplastics significantly affects the plant’s reaction to their presence in the soil. The authors found a positive effect of microplastics on the growth and development of field mustard spinach (*Brassica rapa var. perviridis* L.) in the initial stages of development. However, with an increase in the number of days of exposure to microplastics, significant decreases in fresh and dry biomass (38.51% and 15.12%, respectively) and the yields of fresh and dry edible parts (33.18% and 19.05%, respectively) were found. The influence of the length of exposure to the phytotoxic effects of microplastics on the change in the morphological and biochemical properties of the plant was reported by Adamczyk et al. [87], who studied different concentrations of polybutylene adipate terephthalate (PBAT) plastic on lettuce grown in conditions close to field growing conditions. They measured the observed properties on three occasions—after 4, 8 and 11 weeks of vegetation—and found serious changes in biochemical processes, as well as minor changes in morphological properties. Significant decreases in the shoot height, number of leaves, specific leaf area, and C and N content were recorded at the highest concentration of MPs, while physiological and biochemical processes proved to be more significant indicators of the reaction to the toxic impact of MPs. Specifically, the authors found a significant decrease in the concentration of chlorophyll, an increase in oxidative stress, and significant modifications in the plant’s defense mechanism.

#### 4.3.4. The Impact of Microplastics on Vegetables

Bostan et al. [134] studied the toxic effects of polyethylene film residues on the growth and development of spinach (*Spinacia oleraceae* L.) after the films’ decomposition on the plot. They identified changes in morphological features and biochemical processes due to the toxic action of microplastics. They observed decreases in shoot length (up to 44%), shoot and root fresh mass (63% and 60%, respectively), shoot and root dry mass (54% and 66%, respectively) and leaf surface (up to 40%), while root length was significantly increased. Exposure to MP particles significantly increased the concentration of proline, free amino acids and soluble sugars, as well as the activity of key antioxidant enzymes (POD, SOD and CAT).

Liu et al. [109] studied the influence of two types of MPs (low-density polyethylene—LDPE-MPS; poly (butylene adipate-co-terephthalate)—PBAT-MPS) on the growth and development of pak choi. The plants were grown on soil that had been incubated with these two types of plastic in two concentrations (0.05% and 2%) a year earlier. Shoot fresh weight was significantly reduced in the presence of PBAT-MPS, leading to decreases of 5.83% and 5.79% in shoot fresh weight and shoot dry weight, respectively, compared to the control. A high concentration of PBAT-MPS also reduced the concentration of O^2−^ content in the plant, while a high concentration of the second type of MPs used in this research (LDPE-MPs) reduced the chlorophyll content in the leaf. In addition, a low concentration of PBAT-MPs significantly increased the content of soluble proteins in the plant.

He et al. [113] determined that the concentration of MPs plays a crucial role in the manifestation of harmful effects in plants. They examined the influence of two types of MPs (polyethylene terephthalate; low-density polyethylene) of different sizes and different concentrations on the morphological and physiological properties of peppers (*Capsicum annuum* L.). They found that a concentration of 1% MPs significantly reduced plant weight, fruit yield and antioxidant enzyme activity in leaves and increased the MDA content in leaves, while a significantly lower concentration had a significantly milder negative effect on plant growth. The concentration of microplastics also proved to be the factor that most reduced plant growth and shortened vegetation in the research of Wang et al. [38], who studied the joint toxic effects of MPs of different types (polyethylene—PE; polypropylene—PP) and concentrations (0.1%—PE, PP; 1%—HPE, HPP) and cadmium. The greatest reduction in plant growth was recorded at higher concentrations of microplastics (both types). Increased concentrations of MPs also affected the content of chlorophyll a and b (HPE significantly reduced the concentration of chlorophyll a and b, and HPP significantly reduced the concentration of chlorophyll b, while the concentration of chlorophyll a increased, but this increase was not statistically significant).

Examining the influence of three concentrations (0.02%, 0.2% and 2%) of polylactic acid MPs (PLA-MPs) on the growth and morphological traits of lettuce, Li et al. [105] found that PLA-MP exposure significantly reduced root and shoot length.

A negative effect on the growth and development of plants, as well as changes in physiological biochemical processes, was found by Yar et al. [74], Li et al. [94], Hua et al. [93], Gao et al. [99], Zhang et al. [88,100,102], Yu et al. [111], Chen et al. [107], Han et al. [110] and Pan et al. [79].

Contrary to the previous authors, Cai et al. [89] did not find statistically significant changes in the morphological characteristics or pigment content of lettuce leaves when lettuce containers were exposed to an increased concentration of MPs in the aqueous solution in which the lettuce was grown. The only statistically significant change in morphological characteristics was found in the number of lettuce leaves when MPs were applied at a concentration of 0.05%. Moreover, contrary to most research, López et al. [75] did not find MP particles on broccoli and radish seeds after the seeds were exposed to a microplastic suspension for 24 h, and they concluded that MPs do not affect seed germination. In addition, the same authors found no significant effects of different concentrations of MPs on the root length or shoot length of broccoli seedlings.

As mentioned earlier, the presence of MPs alters the mobility of heavy metals and can enhance their uptake by plants. Liu et al. [84] studied the effect of naturally aged MPs (NAMPs PE—polyethylene) on the mobility of heavy metals (arsenic and cadmium) in soil and their joint effects on lettuce, soil microorganisms and earthworms. They found that although a low concentration of MPs did not have a negative effect on the growth and development of lettuce and did not increase the uptake of arsenic and cadmium, the combined stress of heavy metals and higher concentrations of microplastics (0.5% and 1%) caused a significant decrease in all parameters of root and shoot growth (root and shoot biomass, shoot length, root length) and a 28.4–58.4% decrease in root activity, which stimulated the secretion of low-molecular-weight organic acids (LMVOAs) into the rhizosphere, increasing the bioavailability of As and Cd and increasing their absorption and accumulation in roots and shoots.

Studying the influence of soil properties and the type of MPs on the uptake of heavy metals by plants, Meng et al. [133] found that, in acidic soil, there was increased uptake of Cr, Cu, Pb and Zn and decreased uptake of Cd and Mn, while in alkaline soil, the uptake of heavy metals depended on the type of applied MPs. Thus, ABS and PS MP types increased the uptake of heavy metals, and PS with additives (PA) significantly decreased the uptake of heavy metals. Other treatments (PVC-type MPs without and with additives, ABS and PS with additives) significantly increased the uptake of As, Ba, Cr, Fe, Ni, Pb and Zn, while the application of these treatments reduced the accumulation of Mn in radish. Wang et al. [40] studied the individual effects of three concentrations of polyethylene (PE) MPs (0.1%, 1% and 10%) on the properties of lettuce, as well as the effect of MPs on the mobility and uptake of cadmium, and found that the presence of MPs significantly increased the uptake and accumulation of cadmium. Wang et al. [114] also found statistically significantly higher concentrations of cadmium in the leaves of potherb mustard (*Brassica juncea var. multiceps*) under the influence of different concentrations of MPs. They found that the increase in Cd accumulation increased with the increase in the concentration of MPs.

Changes in the morphological characteristics of lettuce due to the joint action of MPs and heavy metals (As) were also studied by Liu et al. [90]. They applied three different concentrations of As in combination with naturally aged MPs (NAMPs) and identified an antagonistic effect of MPs and heavy metals: at low and medium concentrations of As, there was a decrease in the concentration of As in the shoots and roots of lettuce. In addition, when combining As at these two concentrations (low and medium) with NAMPs, the total biomass of lettuce increased (68.9% and 55.4%, respectively). At the highest applied concentration of As, a joint toxic effect of As and MPs was observed, which manifested in decreases in the aboveground biomass and root mass of lettuce, the concentration of chlorophyll (chlorophyll a and total chlorophyll) in the leaves and the quality of lettuce (total content of soluble sugars and proteins). The content of soluble sugars in the leaf was significantly reduced in the treatment in which the medium concentration of As was combined with NAMPs.

Wang et al. [103] examined the joint toxic effect of Cd and MPs (0.2 µm and 5 µm) applied foliarly and applied to the hydroponic solution in which lettuce was grown. Significant differences in Cd accumulation were observed in the roots, regardless of the way MPs were taken up (via roots or leaves) and regardless of MP size. When using an MP size of 0.2 µm and exposing the roots to MPs, a statistically significant increase in Cd concentration in the roots was recorded, and in the case of foliar application, there was a significant decrease in the Cd concentration in the roots. When using MPs with larger dimensions (5 µm), the accumulation of Cd in the roots statistically significantly decreased if MPs were absorbed through the roots, whereas it significantly increased with foliar application. These opposite trends suggest that both the particle size and exposure pathway modulate the interaction between MPs and Cd uptake in plants.

Dong et al. [77] found that with the combination of arsenic and microplastics, the plastic particles are not only retained in the intercellular space of carrot roots, as is the case with the MP-only treatment, but also transported into cells. They also found plastic particles in the leaves.

The enhanced uptake and toxic effects of heavy metals in the presence of microplastics were also reported by Xu et al. [91], Roy et al. [118], Men et al. [106], Bethanis and Golia [97] and Xu et al. [95].

Although many authors have found that heavy metals have more toxic effects in the presence of microplastics, Li et al. [92] obtained the opposite results regarding the morphological characteristics of pak choy. They studied both the individual and combined effects of three different concentrations of MPs (0.5%, 1% and 2% polyethylene type (PE)) and two concentrations of cadmium (3 mg/kg and 6 mg/kg) on soil and pak choy properties and found no significant difference in the mass of aboveground and belowground biomass between the control variant and all treatment variants. In agreement with the majority of research, these authors found a decrease in chlorophyll content when simultaneously treating plants with increased concentrations of cadmium and MPs.

Similarly to the previous authors, Wang et al. [83] studied the combined phytotoxic effects of Cd and different types of MPs (polyethylene—PE; aged polyethylene—aged PE; polypropylene—PP; aged polypropylene—aged PP; biodegradable polybutylene adipate terephthalate—PBAT; polylactic acid—PLA). They found that pristine PE and PP did not exhibit significant phytotoxicity, while aged PE and PP were highly phytotoxic. In contrast, biodegradable PBAT and PLA promoted root biomass and reduced cadmium concentrations in both soil and plant tissues.

In addition to changes in the absorption of heavy metals, microplastics can affect the absorption of other harmful compounds found in the soil, such as antibiotics or pesticides, which can significantly impair food safety. In order to determine the potential consequences of soil pollution with microplastics for food safety under real conditions of agricultural production, where there is a combination of naturally aged microplastics and pesticides in the soil, Ju et al. [115] studied the influence of microplastics on the bioaccumulation of individual pesticides (chlorpyrifos—CPF; difenoconazole—DIF), as well as their mixture, and their influence on the morphological properties of radish (*Raphanus sativus* L.) in soil containing MPs (low-density polyethylene (LDPE) and biodegradable MPs, pristine and 1 month old). Pristine LDPE MPs led to a 100% increase in fresh root mass, while in the presence of old MPs (1-month LDPE MPs), the fresh root mass of radish decreased by 40%. In the presence of biodegradable MPs (both pristine Bio-MPs and 1-month Bio-MPs), fresh root mass was reduced by 53% and 59%, respectively. The fresh weight of leaves, as well as the ratio of roots to shoots, followed the same change trend as the fresh weight of roots, but the difference was not statistically significant. Root diameter and length were significantly higher in the presence of pristine LDPE MPs, and LI MPs did not affect leaf length. MPs in combination with pesticides did not significantly change morphological characteristics, which indicates that the changes that occurred in radish were mainly due to the presence of MPs. However, by analyzing the content of pesticides in the roots and leaves, it was confirmed that the presence of MPs, especially older MPs, led to the increased accumulation of pesticides by the plant. Significantly higher concentrations were found in the roots in the Mix (pesticide mixture), LDPE + Mix, 1-month LDPE + Mix and 1-month Bio + CPF treatments and in the leaves in the Mix, LDPE + Mix, 1-month LDPE + CPF and Bio + CPF treatments.

Cui et al. [116] studied the influence of MPs on the uptake of antibiotics (oxytetracycline) and their joint toxic effect on radishes. They found that only the PVC-type MPs enhanced the toxic effect of the antibiotic. Specifically, the fresh mass of shoots and roots decreased significantly only under the effect of the PVC-type MPs, while the joint effect of PVC MPs and antibiotics led to a statistically significant reduction in the fresh mass of roots. All three types of MPs (PA, PP and PVC) had a negative effect on the number of radish leaves, and the PVC and PVC-and-antibiotic treatments had a significant negative effect on the ratio of aboveground and belowground mass.

## 5. Conclusions

Microplastics are ubiquitous in the environment. They have been found in water, soil, air and plants, proving that they enter the food chain. In the last 10 years or so, the attention of scientists has shifted to terrestrial ecosystems and to the impact of MPs on the terrestrial living world. Research confirms that MPs change the environment, induce changes in the physical, chemical and biological properties of the soil, and have different effects on plants; these effects range from phytotoxic effects on seed germination, morphological properties of plants, and physiological and biochemical reactions in plants to stimulating or not significantly affecting the growth and development of plants. Contradictory conclusions found in the literature regarding the impact of MPs on the living world, including on plants, can be attributed to the numerous forms of MPs in nature (fibers, foam, films, etc.), as well as differences in their chemical composition, additive content, shelf life and concentration and their varying interactions with different plant species and different types of soil.

Although more attention has recently been paid to the effect of MPs on crops, most of the research has been conducted under laboratory conditions, and only certain traits are monitored; crop yield is not the main focus of researchers, and studies do not include all plant cultures that are used, either for human consumption or as animal feed. The duration of most research carried out in this area is much shorter than the time during which plants are realistically exposed to MPs in the production process, so these experiments are not sufficient to draw conclusions. Therefore, extensive experimental research in long-term and realistic outdoor conditions is necessary.

In addition, limited research has focused on MPs that are already present in fresh vegetables and other foods on the market, which people consume every day. It is necessary to intensify this research, because the consumption of food that contains plastic particles poses a risk to human health.

## Figures and Tables

**Table 1:** Search queries used for searching in the Scopus database.

Database	Search Strategy	Number of Articles
Scopus	TITLE-ABS-KEY (microplastic* OR “micro-plastics” OR nanoplastic*) AND TITLE-ABS-KEY (vegetables) AND PUBYEAR > 2020 AND PUBYEAR < 2026	260
Scopus	TITLE-ABS-KEY (microplastic* OR “micro-plastics” OR nanoplastic*) AND TITLE-ABS-KEY (vegetables) AND PUBYEAR > 2020 AND PUBYEAR < 2026 AND (EXCLUDE (SUBJAREA, “MEDI”) OR EXCLUDE (SUBJAREA, “SOCI”) OR EXCLUDE (SUBJAREA, “ENER”) OR EXCLUDE (SUBJAREA, “CENG”)	189

*—is used as a truncation symbol in the Scopus search to retrieve all terms beginning with “nanoplastic”, such as *nanoplastic* and *nanoplastics*.

## Data Availability

As a review article, this manuscript primarily synthesizes existing knowledge and published data from the scientific literature. No original datasets were generated or analyzed for this study. All sources of information and data are fully cited within the article.
